# Evaluation of Heart Rate Assessment Timing, Communication, Accuracy, and Clinical Decision-Making during High Fidelity Simulation of Neonatal Resuscitation

**DOI:** 10.1155/2014/927430

**Published:** 2014-04-30

**Authors:** Win Boon, Jennifer McAllister, Mohammad A. Attar, Rachel L. Chapman, Patricia B. Mullan, Hilary M. Haftel

**Affiliations:** ^1^Department of Pediatrics and Communicable Diseases, University of Michigan, Ann Arbor, MI 48109, USA; ^2^Department of Medical Education, University of Michigan, Ann Arbor, MI 48109, USA

## Abstract

*Objective*. Accurate heart rate (HR) determination during neonatal resuscitation (NR) informs subsequent NR actions. This study's objective was to evaluate HR determination timeliness, communication, and accuracy during high fidelity NR simulations that house officers completed during neonatal intensive care unit (NICU) rotations. *Methods*. In 2010, house officers in NICU rotations completed high fidelity NR simulation. We reviewed 80 house officers' videotaped performance on their initial high fidelity simulation session, prior to training and performance debriefing. We calculated the proportion of cases congruent with NR guidelines, using chi square analysis to evaluate performance across HR ranges relevant to NR decision-making: <60, 60–99, and ≥100 beats per minute (bpm). *Results*. 87% used umbilical cord palpation, 57% initiated HR assessment within 30 seconds, 70% were accurate, and 74% were communicated appropriately. HR determination accuracy varied significantly across HR ranges, with 87%, 57%, and 68% for HR <60, 60–99, and ≥100 bpm, respectively (*P* < 0.001). *Conclusions*. Timeliness, communication, and accuracy of house officers' HR determination are suboptimal, particularly for HR 60–100 bpm, which might lead to inappropriate decision-making and NR care. Training implications include emphasizing more accurate HR determination methods, better communication, and improved HR interpretation during NR.

## 1. Introduction

Timely and accurate heart rate (HR) assessments are central to effective neonatal resuscitation [1]. The International Liaison Committee on Resuscitation's recent consensus statement reaffirms that HR should determine the adequacy of resuscitation and guide medical decision-making and that an increase in HR remains the most sensitive indicator of effective resuscitative measures [2].

Incorrect HR determination may lead providers to initiate inappropriate treatment or fail to implement warranted intervention. This is particularly important for HR between 60 and 100 beats per minute (bpm), due to the need to provide positive pressure ventilation (PPV) for HR below 100 bpm and chest compressions for HR below 60 bpm in the setting of effective PPV [1].

Deficiencies in communication of HR have been documented among neonatologists, fellows, house officers, nurse practitioners, nurses, and respiratory therapists [3, 4]. Studies also show clinical HR determinations can be inaccurate; however, most studies were performed on low risk newborns who did not require resuscitation at the time of their assessment and had heart rates greater than 100 bpm [5, 6]. Other studies evaluated HR assessment accuracy across various ranges using manikins, but these assessments included prompts for when the heart rates should be checked and were not performed during full resuscitation scenarios [7, 8]. A recent study using high fidelity simulation (HFS), in which subjects were instructed to assess and verbalize their HR determination before and after any interventions, also found deficiencies in HR accuracy that could lead to inappropriate actions [9].

This study evaluated the timeliness, communication, and accuracy of house officers' HR determination and their subsequent resuscitation decision-making in the context of neonatal resuscitations during required HFS exercises.

## 2. Methods

We designed standardized simulation scenarios, based on the Neonatal Resuscitation Program (NRP) 5th edition curriculum, by adapting scenarios associated with the SimNewB Advanced high fidelity neonatal simulator (Laerdal Medical, Stavanger, Norway). Features of our standardized cases included the need to assess HR and respiratory effort and required house officers to make decisions regarding performing PPV, endotracheal intubation, and chest compressions. Initial HR for all scenarios varied between 0 and 80 bpm. Participants were oriented to the SimNewB Advanced high fidelity neonatal simulator prior to their sessions. HFS scenarios were then conducted during these sessions, using the simulator as an assessment tool.

Each scenario was recorded digitally via a webcam placed on the resuscitation cot to facilitate review. Participants were aware of recording and their faces were not in the field of view. Participants were informed prior to participation in this educational curriculum that these recordings might be reviewed for educational purposes, quality improvement, and research. Our Institutional Review Board provided exempt status for this educational intervention.

The NRP Megacode checklist and a previously established scoring instrument have both been cited in the literature, with several reports evaluating their validity [10, 11]. Elements from this checklist and scoring tool were combined and used to track defined house officer performance characteristics during these resuscitations, including measures related to the timing, accuracy, and communication of HR determination. Two of the instructors (WB and JM) underwent a process to confirm interrater reliability. This process included review and discussion of HR scoring definitions, followed by independent scoring of 5 resuscitation scenarios. The two raters agreed on 117 of 120 (97%) of HR scores. Videos of all recorded scenarios from January through December 2010 were then reviewed by one of these instructors. Data were subsequently deidentified for statistical analysis.

All trainees rotating through the University of Michigan's level 4 NICU from January through December 2010 participated in 1-2 simulated resuscitation sessions per month as part of the monthly rotation curriculum. A session was composed of two different NRP scenarios, in which each HO played the role of NRP team leader in one scenario and the role of the assistant in the other scenario. All house officers were NRP trained and certified prior to their NICU rotations. 82 house officers participated in this study. We analyzed data from 80 of these house officers after limiting the dataset to scenarios that (1) represented the participants' first HFS (in order to represent performance prior to training and performance debriefing) and (2) were not affected by simulation, equipment or video malfunctions, or other participants (Table 1). All house officers (including emergency medicine residents) at the University of Michigan are NRP certified at the start of residency and undergo an NRP recertification process every two years thereafter while in training.

We evaluated HR assessments at three key time points during NR: (1) heart rate assessment #1: initial HR check at birth, (2) heart rate assessment #2: HR check to assess response to resuscitative measures beyond the initial steps of warming/drying/stimulating the infant (i.e., after initiation of PPV), and (3) heart rate assessment #3: HR check after continued resuscitative measures. The simulator's actual HR, trainee's method of HR assessment (auscultation, palpation, or both), timeliness, communication to the lead resuscitator, and accuracy of each HR check were tracked. The appropriateness of decision-making based on NRP defined guidelines was evaluated for each HR assessment. Subsequent HR determination trends and associated decision-making beyond these initial three checkpoints were reported in aggregate when appropriate.

HR checks were defined as timely if they were performed within the first 30 seconds of life (for initial HR checks) or performed after 30 seconds of subsequent intervention based on NRP guidelines (for all subsequent HR checks). HR communication was deemed appropriate if it was reported as a specific numerical rate or within one of the NRP defined HR ranges. HR communication was deemed inappropriate if a specific HR or HR range was not explicitly stated (e.g., stating that the HR was “okay” was not acceptable) or if there was a delay >15 seconds in reporting the HR to the lead resuscitator. HR checks were defined as accurate only if they were communicated as either a specific numerical rate or NRP defined HR range and if the HR the resident reported matched the simulator's set HR. HR checks were deemed inaccurate if they were not communicated as a specific HR or HR range or if the stated HR or range did not conform to the simulator's set HR. Decision-making was deemed correct if the appropriate intervention (per NRP 5th edition guidelines) was performed, based on the simulator's set HR and clinical status at the time of the HR assessment.

The proportion of cases congruent with NR guidelines was calculated for HR determination timing, communication, accuracy, and subsequent decision-making. We further evaluated HR ranges relevant to decision-making in NR: <60, 60–99, and ≥100 bpm. Chi square analysis examined the distribution of correct performance across HR ranges with significance set at *P* < 0.05.

## 3. Results

Table 2 summarizes trainees' performance on the defined HR assessments. 72% of all HR assessments were performed via palpation of the umbilical cord, with 13% via auscultation. Both methods were used in 15% of HR assessments. 82% of all HR checks occurred in a timely manner. The first HR assessment was initiated within 30 seconds in 57% of cases, with an average initiation time of 37 seconds. Timing of the first HR check was as early as 4 seconds and as delayed as 115 seconds. 74% of all HR assessments were communicated appropriately to the team leader. 10% of trainees communicated HR with nonspecific language (i.e., without explicit numerical values or ranges).

70% of all HR determinations were accurate within the appropriate NRP defined range. For the first HR assessment, medical decisions consistent with NRP guidelines occurred in 91% of cases. Decision-making was correct with 81% of all HR checks. Excluding the first HR assessment, appropriate decisions were made 75% of the time.

Chi square analysis (Table 3) revealed statistically significant differences in the timeliness, communication, and assessments among the three NRP defined HR ranges: <60 bpm (range 1), 60–99 bpm (range 2), and at least 100 bpm (range 3). In contrast, the appropriateness of decision-making did not vary significantly among the defined HR ranges.

HR was assessed in a timely manner: 90% of the time for HR range 1 (<60 bpm), 75% of the time for HR range 2 (60–99 bpm), and 91% of the time for HR range 3 (≥100 bpm). 87% of HR in range 1 was determined accurately, while HR in range 2 was accurately assessed 57% of the time, and HR in range 3 was accurate in 68% of cases.

Certain performance metrics appeared to be worse in PGY2 residents, who would have been the furthest from initial training or NRP renewal. For example, HR was measured correctly by first-year residents 78% of the time and by third-year residents 69% of the time but by second-year residents only 61% of the time. Appropriateness of medical decision-making improved slightly with increased years of training, with 83%, 87%, and 89% of first-, second-, and third-year residents, respectively. making the correct next step. There were otherwise no statistically or clinically significant differences in any of the measured categories when compared across HO training programs, by residency year, or number of months of NICU experience.

## 4. Discussion

Timely and accurate heart rate determination, communication, and interpretation are central to effective neonatal resuscitation. Our study of residents' observed behavior with HFS in the neonatal setting provides empirical insights into important limitations of their practice with direct implications for training. Auscultation of the HR with a stethoscope is more accurate than palpation of the umbilical cord [5, 6]. In our study of house officers participating in HFS of neonatal resuscitations, 72% of HR checks were done via palpation only, while 13% were auscultated with a stethoscope only and 15% used both auscultation and palpation.

We analyzed the first and subsequent HR evaluations separately because of the possibility of improved HR assessment with subsequent HR evaluations and because the required intervention may vary between the first and subsequent measured HRs. Despite delays in the first HR check, we found that 91% of cases still provided correct initial medical intervention. This discrepancy suggests that, in practice, the first HR check may not be as integral to initial decision-making in the resuscitation scenarios, particularly if the infant appears depressed to the resuscitation team (e.g., the infant is apneic). Correct decisions were made 75% of the time in association with all HR checks when excluding the initial HR check. This latter figure may more accurately reflect the assessment and use of HR during ongoing neonatal resuscitation.

Our findings that HR checks were not consistently communicated are congruent with existing research [3, 4, 12]. Communicating HR with nonspecific terms such as “good” or “okay” may be misinterpreted by other team members, leading to incorrect interventions that are range specific.

Among HR visibly tapped in video review, only 56% were accurate. There were also instances in which tapping was performed correctly (i.e., it matched the simulator's programmed HR) but the stated HR was inaccurate. HR determination in the midst of a stressful resuscitation is prone to error; this may be complicated by the need to multitask counting the heart beat while keeping track of the time spent counting, followed by calculations to derive an actual rate. Trainees inconsistently used the 6-second method of counting HR recommended by the NRP. Counting devices have been proposed to help simplify this process, but they are not widely available [7, 13].

70% of all HR assessments in our sample were accurate within NRP defined ranges. We found clinical HR determination least accurate between 60 and 100 bpm (57% accuracy rate). Errors in HR determination, particularly in the critical range between 60 and 100 bpm, may lead to inappropriate resuscitative measures, such as the provision of chest compressions when they are not indicated or failure to provide PPV when it is indicated. Our findings corroborate the results reported by Chitkara et al. [9] on NRP trained subjects, in which 22 out of the 67 subjects were pediatric residents. Their randomized controlled study using simpler full resuscitation scenarios, which evaluated the accuracy of HR determination, found an error rate of 36% to 48% for rates of 90 bpm, which is similar to the 43% rate our study identified. Junior residents take the task of HR evaluation in most of the neonatal resuscitations in our institution. Neonatology fellows and attending physicians quickly take up this task when the HR is reported to be lower than 100 bpm, given the potential inaccuracy of trainees' HR determinations.

Previous studies [7, 8] evaluating the accuracy of HR assessments using manikins found deficiencies in HR assessment accuracy. However, those assessments were performed in limited scenarios (i.e., not full neonatal resuscitation), and participants in those studies received prompts to assess and communicate HR appropriately. Our study builds on this existing research through the use of full resuscitation scenarios, during which HR assessments were not prompted.

The 6th edition of the NRP recommends use of pulse oximetry (PO) during neonatal resuscitation [14]. In addition to providing saturation values, PO allows continuous HR assessments for infants receiving prolonged resuscitation [15]. More recent studies have found that electrocardiogram (ECG) provides accurate HR information faster than PO [16, 17]. The ability to obtain accurate HR assessments quickly with technology such as ECG or PO may in part ameliorate the deficiencies in clinical HR assessments we have found in this study. Regardless of which technology is used, the ability to have ongoing HR assessments during resuscitation would allow members of the team to perform other tasks and possibly lead to better outcomes. Our findings support the NRP's 6th edition recommendation to utilize objective heart rate monitoring technology early in resuscitation. If the recommendations change practice, this may render this study's findings less relevant in the years to come with the increased use of PO or ECG early during resuscitation. However, both the common finding that medical practice does not always quickly adopt recommended practices and the potential for limited access to the recommended technology in all delivery situations suggest that clinical assessment remains an important skill.

There was a tendency towards worse performance metrics in the PGY-2 group. Almost half of that group (14/29) were emergency medicine residents. However, these emergency medicine residents had similar intervals as the other residents from their last NRP certification. Our findings suggest that reinforcement before the two-year recertification process may be beneficial. House officer training could potentially be improved with educational interventions such as a HFS curriculum. However, follow-up studies of HO performance upon repeated exposure to such a curriculum would be needed to assess this further.

This study used HFS, with data obtained via video review of resuscitation cases. While this manikin represents muscle tone, heart rate, respiratory effort, cyanosis, and cry, assessment nevertheless only approximated that of a real infant. Equipment was subject to malfunction, which was responsible for the exclusion of 7 scenarios. Audiovisual review was limited to what could be recorded clearly. Furthermore, this study does not establish a causal relationship between HR determination/accuracy and clinical decision-making but rather makes available empirical assessments of their frequency and associations.

## 5. Conclusion

Clinical HR determination by house officers during simulated neonatal resuscitation is suboptimal and appears to be least accurate between 60 and 100 bpm. Errors in HR determination, communication, or interpretation may lead to inappropriate resuscitative measures and adverse outcomes. Additional educational emphasis beyond what is provided in current training should be undertaken to improve the clinical acquisition and use of HR during neonatal resuscitation.

## Figures and Tables

**Table 1:** Medical training level and specialty of house officer participants in the baseline high fidelity neonatal resuscitation simulation exercises.

Residency training level	HO 1	HO 2	HO 3	HO 4	Total
Pediatrics	28	9	12	—	49
Medicine/pediatrics	7	6	3	1	17
Emergency medicine	0	14	0	0	14

**Table 2:** House officers' percentage of correct performance on heart rate (HR) assessments and clinical decision-making on neonatal high fidelity simulation resuscitations.

	Overall	First heart rate assessment	Second heart rate assessment	Third heart rate assessment	Subsequent heart rate assessment
HR assessment method					
Palpation	72%	66%	72%	71%	77%
Auscultation	13%	8%	14%	15%	14%
Both	15%	25%	14%	14%	9%
Timely HR assessment	82%	57%	90%	94%	82%
HR communicated appropriately	74%	81%	75%	74%	65%
HR accurate	70%	74%	70%	64%	70%
Clinical decision-making for HR	81%	91%	81%	73%	80%

**Table 3:** House officers' performance on high fidelity neonatal simulation heart rate assessment measures according to actual heart rate.

	HR < 60 bpm	HR 60–99 bpm	HR ≥ 100 bpm	*P* value
Heart rate assessed in a timely manner	90%	75%	91%	*P* < 0.001
Heart rate communicated appropriately	85%	76%	70%	*P* = 0.002
Heart rate accurate	87%	57%	68%	*P* < 0.001
Correct decision-making	82%	80%	86%	*P* = 0.357
